# The Physical Character of Teeth in Consumptive Persons

**Published:** 1857-01

**Authors:** 


### ARTICLE XVI.

### *The Physical Character of Teeth in Consumptive Persons.*

Dr. Thompson, in a lecture delivered before the Medical Society of London, observes, that in a large proportion of individuals affected with phthisis, at a very early period of the disease, and even in the members of consumptive families, amongst whom distinct manifestations of the malady have not yet appeared, you may observe a peculiar, transparent appearance of the edge of the gums, where they are reflected over the teeth, apparently resulting from fineness of structure; in other instances, especially if erethism of the mucous membrane exist, you may observe a border of lake or vermilion tint, such as we might suppose to originate from congealed blood in the capillaries attracting oxygen with great activity.

Three years since, when my attention was first directed to this inquiry, I observed this appearance in twenty out of twenty-one consecutive cases of phthisical males, and in thirteen of twenty-one phthisical women. More recently, on repeating the investigation, the records being kept by a different assistant, thirty of forty-three women, and ninety-two of one hundred men exhibited one or other of the modifications of gingival border which have been described. I have pursued the inquiry to some extent as respects this condition of the gums in other diseases, and have hitherto rarely observed it, except in persons who either were the subjects of phthisis or who have subsequently become so. The wax model which I now show you represents this appearance in a member of a consumptive family. It preceded the occurrence of cough, and of prolonged expiratory murmur; but the disease has made rapid progress, and is now in the last stage. You may observe that the teeth are perfectly free from tartar. The festoon which I have described differs from the uneven border of redness produced by that source of irritation, although the presence of tartar may doubtless modify the condition occasioned by tubercular affection.

In some doubtful cases, Mr. Harrison did me the favor to remove the tartar; and although the vividness of the border was subsequently in some degree lessened, the appearance remained fully as characteristic as ever. The diffused inflammatory redness resulting from the administration of mercury, or other irritating medicines, is readily distinguishable from the clearly defined border under consideration; and if any similar state should be found in cases of purpura, the concomitant circumstances would probably be adequate to remove any uncertainty regarding the cause. I believe this line to have much diagnostic value in early, or, still more, in threatened phthisis, when unaccompanied by any other morbid condition adequate to explain its occurrence. You perceive that in the model the teeth are represented as fine and delicate. It has sometimes been asserted that the teeth of the consumptive are usually remarkably good. There is a fallacy in this statement. In consumptive persons they are sometimes carious, but usually peculiarly delicate, pearly, or glassy; teeth having the firm, ivory-like aspect characteristic of a healthy condition, being in such persons rare. When you reflect how soon they obtain their characteristic organization in the body, I think you will agree with me that the circumstance thus briefly noted furnishes powerful evidence for the opinion I am endeavoring to illustrate, that phthisis is for the most part related to a constitutional affection, associated with the conditions of early growth.*

* Dr. C. A. Harris, Dr. Robinson and Leunec Coe, all show that caries in the teeth differ in color in consumptive persons, and that an early attention to these organs may be the *means* of detecting and arresting the progress of the disease in its early stages.—ED.

